# Enhancing Corn (*Zea mays* L.) Productivity under Varying Water Regimes with At-Plant Application of Xyway Fungicide

**DOI:** 10.3390/plants13172401

**Published:** 2024-08-28

**Authors:** Isha Poudel, Avat Shekoofa

**Affiliations:** Department of Plant Sciences, University of Tennessee, 2505 E.J. Chapman Dr., Knoxville, TN 37996, USA; ipoudel@vols.utk.edu

**Keywords:** crop yield, irrigation events, water management, Xyway LFR^@FMC^/at-plant fungicide

## Abstract

A fungicide’s ingredients can play a physiological role in crop water-management decisions. Our greenhouse study in 2021 demonstrated that Xyway LFR^@FMC^ at-plant fungicide can significantly improve water-saving potential in corn. In 2022 and 2023, a field study was conducted to validate this finding. The 1.11 L ha^−1^ of Xyway LFR^@FMC^ and no-fungicide/check were the main plot effects. Three water regimes, high (HI) and low (LO) numbers of irrigation events and rainfed (RF), were the subplot effect. Plants treated with Xyway LFR^@FMC^ had significantly higher plant height, stem diameter, and leaf water potential (LWP), and had 11.9, 13.4, and 18.3% higher yield under RF, LO, and HI, respectively, in 2022. In 2023, there were no significant differences for the yield components and growth parameters when the combined effect of fungicide treatments and water regimes was considered. However, plants treated with the fungicide had a higher number of rows per ear, kernel number per row, and cob diameter compared to the check. There was no significant separation for yield among the water regimes in 2023, but the crop yield was overall higher for the fungicide-treated plots. Our results indicate that Xyway LFR^@FMC^ fungicide has the potential to improve plant growth and protect the yield when limited water is applied.

## 1. Introduction

Managing water for crop production is becoming more important as declining groundwater and frequent drought scenarios are becoming common challenges due to climate changes [1,2]. Yield loss due to limitation in water availability is very common in many corn-producing countries around the world, including the United States [3].

The question is how producers can reduce the potential seasonal drought-related damage to their crops. Obviously, they have limited choices to make when comes to water-management practices. According to Waldlander et al. [4], they can modify crop rotations to include less water-intensive crops (such as wheat), employ more water-conserving techniques (like conservation tillage), adopt more effective irrigation equipment, improve irrigation applications, or add supplemental irrigation.

But irrigation is either not available in some parts of the Corn Belt [5] or would require investing in expensive irrigation equipment [6]. Producers in varying regions from semi-arid to sub-humid with access to limited rainfall from June to August often have to choose among options such as only properly irrigating a portion of the field area, deficit-irrigating a larger crop area, switching to crops that require less water, or investing in more efficient irrigation technologies [7,8].

Research has shown that irrigation can increase crop yield [6]. However, scheduling irrigation in sub-humid areas has proven difficult due to the high probability of seasonal rainfall. Since both excessive and insufficient water application can significantly reduce the potential for profitability, irrigation scheduling is necessary to control irrigation for maximum performance [8,9]. Through site-specific variable-rate irrigation (VRI) management of crops, precision agriculture offers the ability to improve irrigation efficiency and can address inefficient irrigation scheduling [10]. Soil water sensors can be used as a supplement to VRI systems to enhance irrigation scheduling [11].

According to West and Kovacs [10], networks of remote soil water sensors efficiently provide information to VRI equipment and improve irrigation efficiency. In order to forecast when water is truly required during critical crop growth stages, soil water sensors can more accurately measure soil water availability in the soil profile [11,12]. Tassel emergence to the start of grain filling are the most crucial growth periods for corn; if water requirements are not satisfied during these phases, the yield would drastically suffer [13,14].

Furthermore, previous research results have identified that some classes of fungicide, such as triazoles and strobilurins, not only alleviate fungal diseases, including gray leaf spot, northern corn leaf blight, and southern corn rust caused by *Cercospora zeae-maydis*, *Setosphaeria turcica*, and *Puccinia polysora*, respectively [15,16], but they also regulate various physiological and biochemical phenomena. They have been found to mediate water-deficit stress and promote plant growth and yield [17,18,19].

Triazole compounds are systemic fungicides that have plant growth-regulating properties and are called plant multi-protectants because of their natural ability to induce abiotic stress tolerance by increasing antioxidant enzymes and molecules in stress-affected plants [20,21]. Triazoles modify the isoprenoid pathway and alter the level of certain hormones by suppressing gibberellin synthesis, decreasing ethylene evolution, and increasing cytokinin levels [20,22]. However, despite these beneficial effects, resistance to triazole fungicides has emerged in several plant fungal pathogens, including *Sphaerotheca fuliginea*, *Puccinia triticina*, *Zymoseptoria tritici*, and *Penicillium digitatum* [23,24].

Triazole treatment modified the biochemical content and antioxidant enzyme activity in okra (*Abelmoschus esculentus* L.) and resulted in a partial recovery of the damaging effect of drought stress by its influence on the antioxidant system [25]. The mixture of strobilurin and triazole family, pyraclostrobin (12.5% *w*/*w*) + epoxiconazole (4.7% *w*/*w*), also called opera, has positive physiological and morphological effects on soybean (*Glycine max* L.) under a water stress environment [26].

A product called Xyway LFR^@FMC^, a novel triazole class of fungicide, is also known as a demethylation inhibitor (DMI) fungicide. Flutriafol is the active ingredient in Xyway brand fungicides, which are systemic in nature. Flutriafol is moderately mobile and so may leach into groundwater under certain conditions, raising the risk of groundwater contamination [27,28,29]. In a study by Aliste et al., triazole fungicides (flutriafol, myclobutanil, and triadimenol) were found to pollute groundwater resources, mainly in areas with intensive yearly rainfall regimes [30]. The persistence of flutriafol in soil, measured as the half-life (t1/2), is 1177.3 days under field conditions and 1587 days in laboratory conditions [31].

In August 2020, FMC Technologies, Inc. introduced this fungicide with the goal of protecting against gray leaf spot, northern corn leaf blight, and other foliar diseases from the root to the tassel and from the stalk to the leaf. Poudel and Shekoofa [17] reported that in a set of greenhouse studies, the application of 1.11 L ha^−1^ of Xyway LFR^@FMC^ fungicide at planting improved the corn plants’ water-saving potential by controlling the transpiration rate and limiting stomatal conductance.

Therefore, a study was initiated to determine the effectiveness of an application of 1.11 L ha^−1^ Xyway LFR^@FMC^ at-plant fungicide under field conditions. The objective of this study was to assess the Xyway LFR^@FMC^ at-plant fungicide’s contribution to any plant’s physiological, growth, and yield responses to three water-regime scenarios in large plots field trials.

## 2. Results and Discussion

A statistical analysis revealed that the fungicide treatments, water regimes, and their interaction effect were statistically significant for the yield and a few other parameters, including cob length and LWP (leaf water potential), in the year 2022 (Table 1). However, the water regimes and interaction effect were not significant for the yield in the year 2023 (Table 1) due to high rainfall events throughout the season.

The effect of water regimes and fungicide treatments on the plants’ height were significant and varied by year (Table 1 and Table 2). In 2022, the corn plants treated with 1.11 L ha^−1^ of Xyway LFR^@FMC^ fungicide under all water regimes consistently resulted in taller plants (Table 2). Corn plants treated with the at-plant fungicide under rainfed (RF) conditions were as tall as the untreated (i.e., without fungicide treatment) plants under a low number of irrigation events (LO) (Table 2).

Sankar et al. [32] reported that when peanut (*Arachis hypogaea* L.) plants were treated with 10 mg L^−1^ of paclobutrazol (a triazole) fungicide under drought stress, the treated plants had longer stems compared to drought-stressed plants without paclobutrazol treatment. Triazole fungicides have been found to improve plant growth, contrary to their growth-retarding effects in some cases [33]. In a study by Ijaz et al. [34], it was reported that triazole and strobilurin fungicides increased the plant height and had positive effects on the growth parameters of winter rapeseed (*Brassica napus* L.).

When plants were treated with fungicide under high (HI) and low (LO) numbers of irrigation events and rainfed (RF) water regimes, their stem diameters were larger than plants without fungicide/check (Table 2). The at-plant fungicide treatment resulted in a significant increase by 4.2 to 5.4% when comparing HI and LO with LO under no fungicide application (Table 2). The RF plants treated with the fungicide had a 2.4% larger stem diameter compared to the similar water regime under no fungicide application (Table 2).

**Figure 1:**
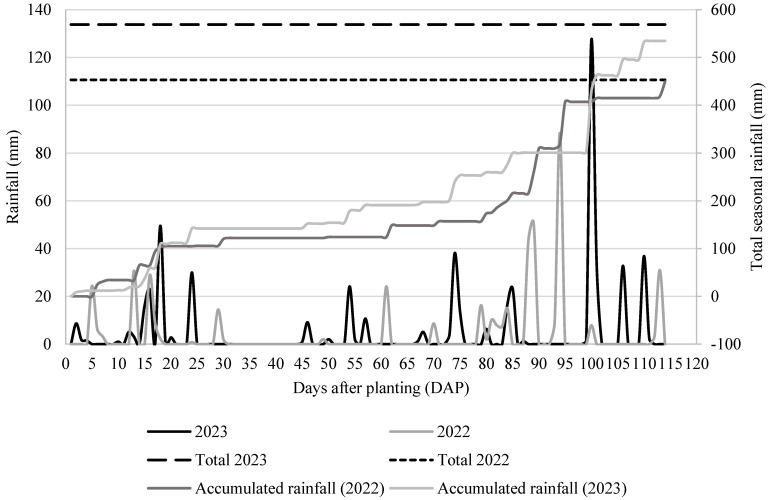
Rainfall (mm) amount in days after planting, total seasonal rainfall, and accumulated rainfall in 2022 and 2023.

In our study in 2023, the plants’ height and stem diameter were not affected by either fungicide treatments or water regimes (Table 2). This could be caused by a shift in rainfall seasonality. In 2022, there were only three rainfall events from 19 to 60 days after planting (Figure 1), which is a critical period for plant growth and development [35,36]. Thus, the change in rainfall patterns may have created the separation between the treatments. Additionally, Pandey et al. (2000) reported that the deficit water availability at the beginning of plants’ vegetative growth phase can affect the leaf area index, plant height, and plant growth rate [37]. In the 2023 season, there was an even distribution of rainfall within this critical period (Figure 1).

In 2022, the water regimes and fungicide treatments significantly impacted the plants’ ear height (Table 2). An analysis of variance (ANOVA) showed that plants treated with the fungicide had 13, 14, and 9% higher ear height under HI, LO, and RF regimes, respectively, compared to plants without any fungicide treatment (Table 2) under the exact same water regimes.

There was no significant difference in the plants’ RWC (%) for either fungicide treatments or water regimes in both years (Table 2). In contrast to our finding, the application of paclobutrazol at (1498.2 mg L^−1^), a triazole compound, kept the plants’ RWC (%) higher than the non-treated plants when subjected to water-deficit stress [38,39]. In their study, Poudel and Shekoofa [19] indicated that corn treated with 1.11 L ha^−1^ Xyway LFR^@FMC^ had significantly less water loss compared to check/no fungicide treatment under water-deficit conditions in the greenhouse. The plants’ shoot hydraulic conductance and LWP were higher at fungicide application rates of 1.11 and 1.26 L ha^−1^ compared to check under water-deficit conditions [19].

**Figure 2:**
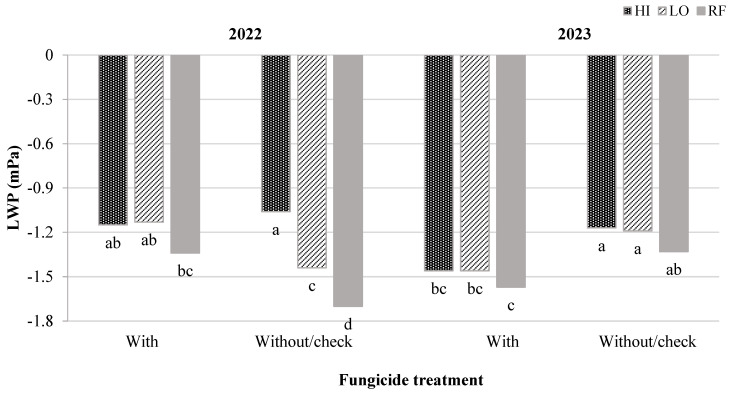
Plants’ LWP (mPa) under two fungicide treatments: without fungicide/check and with 1.11 L ha^−1^ of Xyway LFR^@FMC^, and three water regimes: high (HI) and low (LO) number of irrigation events and rainfed (RF) in 2022 and 2023. Means with different letters are significantly different from one another according to Student’s *t*-test (*p* < 0.05).

In 2022, the interaction effect between fungicide treatments and water regimes was statistically significant (*p* < 0.0145) for LWP. The fungicide-treated plants had improved the LWP by 27.43 and 26.87% compared to plants without fungicide treatment under LO and RF, respectively (Figure 2). This indicates that the fungicide may have reduced water loss through transpiration [19] under water-limited conditions (i.e., rainfed and LO). Thus, the beneficial effect of fungicide application at planting is more evident under water-limited conditions.

Unlike 2022, the LWP values were significantly more negative for the fungicide-treated plants compared to the plants without any fungicide treatment under all water regime scenarios in 2023 (Figure 2). In the 2023 growing season, our soil analysis results confirmed that there was a lower amount of potassium (K^+^) in the fungicide-treated plots area compared to plots without any fungicide treatment (Table 3). Undoubtedly, there is an important role for K^+^ when discussing plants cell membrane stability and osmotic adjustment ability [40]. Hence, the low level of K^+^ in the experimental soil may have influenced the plants’ LWP result in 2023.

In 2022, there was a positive effect of fungicide treatment on the plants’ cob length under all water regime scenarios compared to plants without any fungicide treatment/check. The plants’ cob length amounts were increased by 10, 9, and 4% by the fungicide treatment under HI, LO, and RF, respectively (Table 4).

Plants treated with the fungicide under LO and RF water regimes had 3 to 5% longer ear length, respectively, in 2022 compared to plants without any fungicide treatment/check (Table 4). Furthermore, the fungicide-treated plants under HI and LO water regimes resulted in a significant increase in the cob diameter rates by 3 to 5%, respectively, compared to plants without any fungicide treatment/check in 2022 (Table 4).

A significant difference in the number of rows per ear was observed among the fungicide-treated plants compared to those without any fungicide/check under HI and LO water regimes (Table 4). In 2022, the fungicide treatment increased the number of rows per ear by 17.2, 7.2, and 7.4% under HI, LO, and RF, respectively, compared to the no-fungicide treatment/check (Table 4). In 2023 overall, the fungicide-treated plants had a higher number of rows per ear compared to the no-fungicide treatment/check (Table 4). A possible reason behind the yield component improvements observed in our study might be that the increase in the plants’ growth and canopy area provided the plants with a better condition for light interception and photosynthesis. Zhang et al. [41] reported that azoxystrobin (a strobilurin) and tebuconazole (a triazole) application delayed senescence and hence enhanced the grain yield of winter wheat.

In another study by Berdugo et al. [42], the green leaf area duration was extended by bixafen, fluoxastrobin, and prothioconazole fungicides application compared with the untreated control. They reported that the fungicide treatments numerically increased the number of ears per pot, ear weight, and number of kernels per ear. Delayed senescence/extended green leaf area in plants helps in prolonging the stage of kernel growth and expansion, and as a result, productivity is amplified [42].

No significant difference was observed for kernel protein, oil, and starch in both years (Table 4). In the Ijaz and Honermeier [43] report, the application of various combinations of nine triazole and strobilurin fungicides had no prominent effect on the oil quality of winter rapeseed.

**Figure 3:**
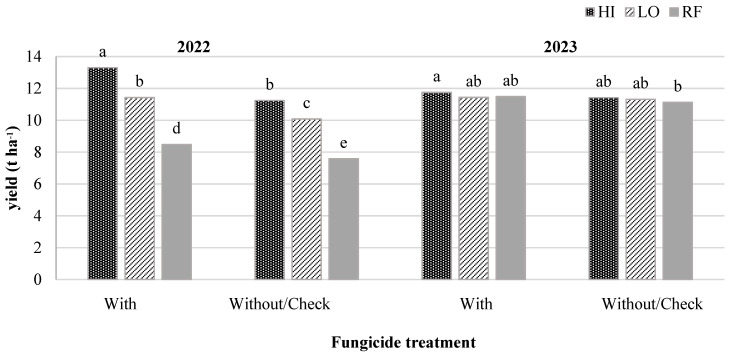
Final grain yield (t ha^−1^) under two fungicide treatments: without fungicide/check and with 1.11 L ha^−1^ of Xyway LFR^@FMC^ and three water regimes: high (HI) and low (LO) number of irrigation events and rainfed (RF) in 2022 and 2023. Means with different letters are significantly different from one another according to Student’s *t*-test (*p* < 0.05).

In 2022, the effect of interaction between fungicide treatments and water regimes on corn grain yield was statistically significant and varied by year (*p* < 0.0483) (Table 1 and Figure 3). Fungicide treatment increased the yield, ranging from 8.5 to 13.3 t ha^−1^ compared to the no fungicide treatment/check under all the water regime scenarios (Figure 3). The application of 1.11 L ha^−1^ of Xyway LFR^@FMC^ at-plant fungicide increased the yield by 11.9, 13.4, and 18.3% under RF, LO, and HI water regimes, respectively (Figure 3).

A substantial number of studies have shown that triazole in combination with strobilurin led to the highest seed yield over other treatments for winter rapeseed (*Brassica napus* L.) [34,44]. A multivariate random-effects meta-analysis was conducted on 12 years of data from 14 U.S. states to determine the mean yield and test-weight responses of wheat (*Triticum aestivum* L.) to treatment with propiconazole, prothioconazole, tebuconazole, metconazole, and prothioconazole + tebuconazole. The study found that all the fungicides led to a significant increase in mean yield and test weight relative to the check [45].

In the 2023 growing season, there were no significant differences observed in the cob length, ear length, cob diameter, kernel number per row, and number of rows per ear when the combined effect of fungicide treatments and water regimes was considered (Table 4). But overall, the plants treated with the at-plant fungicide had numerically higher number of rows per ear, kernel number per row, and cob diameter compared to those without any fungicide treatment (Table 4).

**Figure 4:**
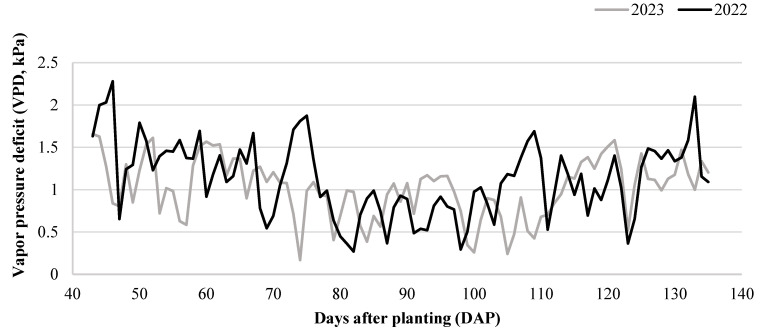
Vapor pressure deficit (VPD, kPa) during days after planting in 2022 and 2023.

The lack of consistency for these observations between the 2022 and 2023 growing seasons data could be explained by sufficient rainfall during early vegetative, pollination, and grain-filling stages (Figure 1). Additionally, the rainfall was more evenly distributed in 2023 versus 2022 (Figure 1), and in contrast with the 2022 season, the medium vapor pressure deficit (VPD) (i.e., 1.0 to 1.5 kPa) was recorded throughout the growing season in 2023 (Figure 4). Favorable weather conditions might be another reason we could not see differences in most growth and physiological parameters and yield components for the interaction between fungicide treatments and water regimes in 2023.

The other confounding factors that could lead to variable yield responses between the two years in our study are the presence (and possibly intensity) of plant diseases. Kelly [46] reported that there was little to no disease observed in 2023 during corn vegetative to early reproductive stages in Tennessee. Thus, less disease incidences in 2023 may have reduced the separation of interactions between fungicide treatments and water regimes.

In a study by Swoboda and Pedersen [47], the application of strobilurin and a triazole (Pyraclostrobin rate of 113 g a.i. ha^−1^ and tebuconazole rate of 95 g a.i. ha^−1^) alone or in combination in soybean did not produce a nonfungicidal physiological effect or associated yield improvement in the absence of foliar disease. In another study, the application of three different commercial triazole-, strobilurin-, or carboxamide-based fungicides during the pre-bloom and bloom stages on soybean disease-free plants in the greenhouse did not affect the physiological traits and crop yield [43].

According to Paul et al. [45], variable yield responses to triazole-based fungicide treatments are the presence (and intensity) of foliar diseases and the physiological effects of the fungicide on the crop yield in the absence of any disease and environmental conditions. Hence, the difference in disease occurrence and severity, as well as environmental conditions, including rainfall and evaporative demand (i.e., VPD), may have created the variation in our results in 2022 and 2023.

## 3. Materials and Methods

### 3.1. Field Description and Experimental Design

Field research was conducted at the Milan Research and Education Center (MREC), Milan, TN, in 2022 and 2023 under a center pivot irrigation system equipped with variable-rate irrigation (VRI) technology. A corn hybrid, DKC64-35 (DKC64-35RIB Brand Blend, DeKalb Genetics corporation, DeKalb, IL, USA) was sown, and fungicide was applied at-plant. Planting was initiated on 10 May 2022 and 26 April 2023 with a planting rate of 88,955 seeds ha^−1^ for both years.

For both years, the soil type for the study site was providence silt loam (fine-silty, mixed, active thermic Oxyaquic Fragiudalfs) (Table 3). On 7 May 2022 and 20 April 2023, composite soil samples were collected from the experimental site locations at a depth of 0 to 15 cm. Ten soil probes of 2.5 cm diameter were randomly collected for each sample to determine the parameters indicated in Table 3. The soil samples were sent to the University of Tennessee Soil, Plant, and Pest Center for analysis. In accordance with the most recent guidelines from the University of Tennessee Soil, Plant, and Pest Center, lime, fertilizer, and pesticide were applied. Each year, a preplant spring application of nitrogen (N) was applied using diammonium phosphate (DAP) and ammonium sulphate at a rate of 50 kg N ha^−1^. At the V4–V6 growth stage, a sidedress application of 200 kg N ha^−1^ was applied to the soil as urea ammonium nitrate (UAN). In 2022, a preplant spring application of phosphorous (P), DAP, and potassium (K), K_2_O_5_ was applied at a rate of 100 kg ha^−1^ each. But in 2023, based on the soil test result, the application of P and K was not recommended.

In both years 2022 and 2023, the treatments were arranged in a split plot with a completely randomized design. Fungicide treatments were the main plot effect, and water regimes were the subplot effect. The subplots sizes were approximately 34 × 31 m. The fungicide treatments included (1) without fungicide/check and (2) 1.11 L ha^−1^ of Xyway LFR^@FMC^, and the three water regimes included a high (HI) and low (LO) number of irrigation events and rainfed (RF)/check (Table 5). Each water regime was replicated 3 times within the fungicide and without fungicide/check plots. Four weeks after planting, five loggers (ZL6-Meter logger, METER Group, Inc. Pullman, WA, USA) equipped with soil water content matric potential sensors, TEROS 21 (Meter Group, Inc. Pullman, WA, USA), and digital rain gauges, ECRN−50 (Meter Group, Inc. Pullman, WA, USA), were installed in each fungicide and check plot, one in each irrigation treatment (except for RF in the check plots). The sensors were installed at a depth of 20 and 66 cm to monitor the soil water content and rainfall and thereby schedule irrigation.

### 3.2. Sensor-Based Scheduling

The irrigation scheduling was designed based on the depth and reading of soil matric potential sensors, as well as the percentage of the crop-rooting zone [8,48]. To determine the actual soil water availability for the corn plants’ rooting zone, the sensor reading at 20 cm depth, which represents the top 50% of the rooting zone, was multiplied by 0.5. The same process was applied to the 66 cm depth, representing another 50% of the rooting zone, and the actual value of available water was calculated, following the procedure explained by Sheldon et al. [8].

Irrigation was initiated when the actual value, measured by a ZL6-Meter logger (METER Group, Inc. Pullman, WA) using soil matric potential sensors at depths of 20 and 66 cm, fell within the range of −65 to −75 kPa [8]. At the end of the growing season, each water regime including HI, LO, and RF was irrigated 14, 9, and 0 times, respectively, in 2022 and 6, 4, and 0 times, respectively, in 2023 (Table 5). In each irrigation event, 12.7 mm of water was applied in 2022, whereas in 2023, 10.41 mm of water was applied in each irrigation event, except for two times where 7.62 mm 15.24 mm of water was applied due to some technical issue within the irrigation system. The rainfall amount (Figure 1) was also incorporated when the irrigation decision was made each time.

### 3.3. Data Collection

#### 3.3.1. During Growing Seasons

In the 2022 growing season, in each plot, five plants were randomly selected within a radius of 9 meters from the center of the plot, and the plant height, leaf number, and stem diameter were monitored and recorded weekly. We also measured the leaf water potential (LWP) at 5, 7, 9, and 11 weeks after sowing (WAS), relative water content (RWC) at 13 and 15 WAS, and ear height at 13, 15, and 17 WAS. In 2023, data were collected similarly to 2022, but the plant height and stem diameter data measurements were taken at 4, 6, and 12 WAS, and ear height, LWP, and RWC at 8 and 12 WAS.

For the RWC, two leaves were randomly selected within a 3 m radius from the center of each plot. According to sampling method explained by Mullan and Pietragalla [49], the top-most fully expanded leaf receiving sunlight was selected. As a sample, a circular leaf portion 15 cm in diameter from the middle part of the leaf was collected using a paper punch (Bira craft, Amazon, Washington, DC, USA). The samples were placed in a sealed plastic bag after harvest. To maintain the turgidity of samples, they were immediately kept in a cooler. The samples were taken to the lab to measure the fresh weight (g). After measuring the fresh weight, the samples were placed in a tub of deionized water. The tub with the samples was then exposed to 4 °C in a walk-in cooler. After 6–8 h, the samples’ turgid weights (g) were recorded.

Then, to document the samples’ dry weight, all the samples were placed in individual paper bags (model no. S-7798, Uline.com) and moved to an oven at 80 °C for 12 h. The leaf dry weight (g) was collected soon after the samples were out of the oven. After all the measurements were taken, the following equation was used to calculate the RWC:

$$RWC=\frac{Fresh\;weight-Dry\;weight}{Saturated\;weight-Dry\;weight}\times 100$$

A pressure chamber, model 1505D (PMS Instrument Company, Albany, OR, USA), was used to measure the LWP. For the LWP measurement, each time, two healthy and fully developed leaves were selected per plot. A section of the leaf 10–20 cm from the tip of the leaf blade was cut, and the cut leaf was placed in a plastic bag where the cut end of the leaf blade was positioned outside of the plastic bag. Then, the cut end of the leaf blade was placed in the pressure chamber while the cut end extended outside the chamber and was exposed to atmospheric pressure. The pressure was increased around the leaf until xylem sap appeared at the cut end of the leaf, and then, the pressure was recorded [50,51].

#### 3.3.2. At Harvest

Each plot within a 9.1 m radius from the center of the plot was harvested mechanically on 22 September 2022 and 2023. Before mechanically harvesting the plot, 10 ears were collected within a 3 m radius from the center of the plot, then the cob length, cob diameter, ear length, number of rows per ear, and kernel per row data were documented. Additionally, the kernel oil, protein, and starch were recorded using a near-infrared reflectance diode array feed analyzer (Perten, Springfield, IL, USA).

### 3.4. Statistical Analysis

All the growth, physiological parameters, yield components, and yield data were analyzed using JMP (version Pro 17, SAS Institute Inc., Cary, NC, USA). Two-way ANOVA was employed to analyze the effect of the fungicide levels, irrigation regimes, and their interaction on all the physiological, morphological, and yield components data. The mean values for the treatments with significant effects were subsequently separated using the LSD test. Additionally, to ensure the robustness of our results, a Student’s *t*-test was also conducted to verify the significance of the treatments at *p* < 0.05 level.

## 4. Conclusions

The Xyway LFR^@FMC^ at-plant fungicide application in 2022 improved corn plant height, stem diameter, leaf water potential (LWP), and most yield components under all the water regime scenarios compared to the no-fungicide treatment. Moreover, the fungicide treatment increased the corn grain yield by 11.9, 13.4, and 18.3% under RF, LO, and HI water regimes, respectively, in 2022. However, in 2023, the environmental conditions were markedly different. The frequent rainfall, low disease pressure, and a vapor pressure deficit (VPD) range of 1.0 to 1.5 kPa impacted our results. These favorable conditions reduced the benefit of the fungicide treatment. While there were no significant interactions between the fungicide treatment and water regimes in 2023, the impact of the fungicide application at planting was statistically significant on the yield. Our 2022 data clearly indicate the positive effects of Xyway LFR^@FMC^ at-plant fungicide applied at the rate of 1.11 L ha^−1^ on improving corn morphology, physiology, and yield under varying water regime scenarios, but the weather and environmental conditions in 2023 limited the ability to draw similar conclusions. Thus, to address the varying environmental conditions and further validate our findings and provide a more comprehensive assessment, the use of crop modeling practices can be pursued.

## Figures and Tables

**Table 1:** Overall effect of fungicide treatments, water regimes, and their interactions on yield, yield-attributing parameters, and nutritional characteristics of corn in 2022 and 2023.

Parameters	Source of Variation	Fungicide Treatments	Water Regimes	Fungicide Treatments × Water Regimes
	Df	1	2	2
**2022**				
**Yield (t ha^−1^)**	Sum of Squares	6158.30	36779.00	685.54
	*p*-value	<0.0001 ***	<0.0001 ***	0.0483 **
**Plant height (cm)**	Sum of Squares	8904.71	21429.54	1507.19
	*p*-value	<0.0001 ***	<0.0001 ***	0.1281 ^ns^
**Stem diameter (cm)**	Sum of Squares	0.13	0.72	0.05
	*p*-value	0.0009 ***	<0.0001 ***	0.1129 ^ns^
**Ear height (cm)**	Sum of Squares	1216.33	846.41	60.48
	*p*-value	<0.0001 ***	<0.0001 ***	0.2853
**Cob length (cm)**	Sum of Squares	41.60	69.30	27.64
	*p*-value	<0.0009 ***	<0.0001 ***	0.0241 **
**Cob diameter (cm)**	Sum of Squares	17.52	8.63	5.46
	*p*-value	0.0027 ***	0.1045 ^ns^	0.2374 ^ns^
**Kernal number per row**	Sum of Squares	5.71	6.10	1.97
	*p*-value	0.1136	0.2612	0.6466
**Number of rows per ear**	Sum of Squares	311.41	227.15	61.48
	*p*-value	0.0001 ***	0.0038 ***	0.2118
**Ear length (cm)**	Sum of Squares	9.50	45.99	12.40
	*p*-value	0.1056	0.0022 ***	0.1808
**LWP (mPa)**	Sum of Squares	0.68	2.09	0.72
	*p*-value	0.0049 **	<0.0001 ***	0.0145 **
**RWC (%)**	Sum of Squares	0.08	13.67	31.32
	*p*-value	0.9595 ^ns^	0.7951 ^ns^	0.5928 ^ns^
**Protein content (%)**	Sum of Squares	0.0071	4.52	0.94
	*p*-value	0.8948 ^ns^	0.0233 **	0.3387 ^ns^
**Oil content (%)**	Sum of Squares	0.0	0.11	0.18
	*p*-value	0.3558 ^ns^	0.4347 ^ns^	0.2934 ^ns^
**Starch content (%)**	Sum of Squares	0.30	10.21	0.87
	*p*-value	0.2733 ^ns^	0.0003 ***	0.1967 ^ns^
**2023**				
**Yield (t ha^−1^)**	Sum of Squares	280.89	191.56	49.90
	*p*-value	0.0413 **	0.2353 ^ns^	0.6814 ^ns^
**Plant height (cm)**	Sum of Squares	90.20	1441.11	86.07
	*p*-value	0.7007 ^ns^	0.3084 ^ns^	0.9317 ^ns^
**Stem diameter (cm)**	Sum of Squares	0.07	0.09	0.02
	*p*-value	0.1731 ^ns^	0.3030 ^ns^	0.7336 ^ns^
**Ear height (cm)**	Sum of Squares	84.49	47.42	15.29
	*p*-value	0.0188 ***	0.2060	0.5969
**Cob length (cm)**	Sum of Squares	0.90	28.71	0.34
	*p*-value	0.4821 ^ns^	<0.0001 ***	0.9002 ^ns^
**Cob diameter (cm)**	Sum of Squares	3.69	2.54	0.13
	*p*-value	0.0664	0.3116	0.9437
**Kernal number per row**	Sum of Squares	34.97	307.28	0.26
	*p*-value	0.1567	0.0002 ***	0.9924
**No. of rows per ear**	Sum of Squares	0.04	2.33	1.66
	*p*-value	0.8811 ^ns^	0.5513 ^ns^	0.6527 ^ns^
**Ear length (cm)**	Sum of Squares	5.88	15.75	1.32
	*p*-value	0.0943	0.0247 **	0.7272
**LWP (mPa)**	Sum of Squares	1.25	0.28	0.08
	*p*-value	<0.0001 ***	0.0741 ^ns^	0.9298 ^ns^
**RWC (%)**	Sum of Squares	92.97	2506.47	71.19
	*p*-value	0.5840 ^ns^	0.0220 **	0.8906 ^ns^
**Protein content (%)**	Sum of Squares	0.11	0.15	0.09
	*p*-value	0.4007 ^ns^	0.6164 ^ns^	0.7356 ^ns^
**Oil content (%)**	Sum of Squares	0.05	0.03	0.0021
	*p*-value	0.3065 ^ns^	0.6738 ^ns^	0.9796 ^ns^
**Starch content (%)**	Sum of Squares	0.03	0.31	0.12
	*p*-value	0.8838 ^ns^	0.8363 ^ns^	0.6482 ^ns^

Df: Degree of freedom. *** and ** indicate level of significance at 1 and 5% respectively. ^ns^: not significant. Abbreviations: LWP, leaf water potential; RWC, relative water content.

**Table 2:** Plants’ height, stem diameter, relative water content (RWC), and ear height under two fungicide treatments: without fungicide/check and with 1.11 L ha^−1^ of Xyway LFR^@FMC^, and three water regimes: high (HI) and low (LO) number of irrigation events and rainfed (RF) in 2022 and 2023.

Fungicide Treatment	Water Regime	Height (cm)	Stem Diameter (cm)	RWC (%)	Ear Height (cm)
**2022**					
**With**					
	**HI**	247.14 a	2.36 a	91.71 ^ns^	123.62 a
	**LO**	227.08 b	2.39 a	89.24	114.30 b
	**RF**	201.42 c	2.18 b	91.41	107.37 c
**Without/check**					
	**HI**	219.56 b	2.34 a	90.89	108.20 bc
	**LO**	205.66 c	2.26 b	91.12	99.24 d
	**RF**	192.76 c	2.13 c	90.14	98.22 d
**2023**					
**With**					
	**HI**	102.44 ^ns^	1.91 ^ns^	81.20 a	87.96 abc
	**LO**	89.22	1.78	78.89 ab	84.46 c
	**RF**	99.92	1.80	66.84 ab	85.17 bc
**Without/check**					
	**HI**	100.15	1.93	77.05 ab	93.47 a
	**LO**	80.62	1.85	73.48 ab	91.62 ab
	**RF**	99.85	1.91	65.15 b	87.31 abc

Means followed by different letters within columns indicate significant difference at *p* < 0.05 Student’s *t*-test for pairwise comparison. ^ns^: not significant.

**Table 3:** Soil properties of the research plots at Milan Research and Education Center (MREC) for 2022 and 2023.

Fungicide Treatment	pH	Phosphorus (ppm)	Potassium (ppm)	Calcium (ppm)	Magnesium (ppm)	Soil Type
**2022**						
**Without/check**	6.30	24.5	96.5	2034.0	81.5	Providence silt loam (fine-silty, mixed, active thermic Oxyaquic Fragiudalfs)
**With**	6.20	25.0	103.0	2226.5	75.0	Providence silt loam (fine-silty, mixed, active thermic Oxyaquic Fragiudalfs)
**2023**						
**Without/check**	6.60	17.0	74.0	1363.0	69.0	Providence silt loam (fine-silty, mixed, active thermic Oxyaquic Fragiudalfs)
**With**	6.80	24.0	59.0	1345.0	71.0	Providence silt loam (fine-silty, mixed, active thermic Oxyaquic Fragiudalfs)

**Table 4:** Plants’ yield components measurements under two fungicide treatments: without fungicide/check and with 1.11 L ha^−1^ of Xyway LFR^@FMC^, and three water regimes: high (HI) and low (LO) number of irrigation events and rainfed (RF) at harvest in 2022 and 2023.

Fungicide Treatment	Water Regime	Cob Length (cm)	Ear Length (cm)	Cob Diameter (cm)	Kernel Number per Row	Number of Rows per Ear	Protein Content (%)	Oil Content (%)	Starch Content (%)
	**2022**								
**With**									
	**HI**	18.57 a	22.94 ab	2.44 a	17.19 ^ns^	32.82 a	10.00 abc	3.47 a	62.10 ab
	**LO**	18.56 a	23.60 a	2.47 a	17.32	31.60 ab	9.70 bc	3.37 a	62.20 ab
	**RF**	16.30 b	22.52 b	2.36 b	16.74	28.70 c	11.03 a	3.70 a	60.43 c
**Without/check**									
	**HI**	16.83 b	23.28 ab	2.36 b	17.00	28.00 c	9.35 c	3.20 a	63.00 a
	**LO**	16.98 b	22.82 ab	2.36 b	16.59	29.46 bc	10.30 abc	3.55 a	61.90 b
	**RF**	15.64 c	21.43 c	2.35 b	16.44	26.72 c	10.95 ab	3.40 a	60.70 c
	**2023**								
**With**									
	**HI**	17.53 a	23.35 a	2.44 a	33.45 a	16.14 ab	9.40 ^ns^	3.25 ^ns^	61.65 ^ns^
	**LO**	16.55 b	22.37 b	2.43 ab	29.59 cd	16.20 ab	9.63	3.30	61.33
	**RF**	17.42 a	22.88 ab	2.40 ab	31.76 abc	15.93 abc	9.45	3.35	61.60
**Without/check**									
	**HI**	17.44 a	22.68 ab	2.40 ab	32.32 ab	15.19 d	9.13	3.13	61.53
	**LO**	16.47 b	22.14 b	2.40 ab	28.63 d	15.47 bcd	9.33	3.17	61.60
	**RF**	17.33 a	22.67 ab	2.37 b	30.83 bcd	15.32 cd	9.50	3.27	61.53

Means followed by different letters within columns indicate significant difference at *p* < 0.05 Student’s *t*-test for pairwise comparison. ^ns^: not significant.

**Table 5:** Irrigation scheduling during 2022 and 2023 field experiment.

Week	Growth Stage	High (HI) Number of Irrigation Events	Low (LO) Number of Irrigation Events	Rainfed (RF)
**2022**				
**5–7**	V7-tassling	6× *	4×	0
**8–9**	Ear size potential/silking to blister	4×	2×	0
**10–11**	Grain filling/soft dough	4×	3×	0
**Total irrigation applied (mm)**		177.8	114.3	0.0
**2023**				
**9–10**	Silking to blister	3×	3×	0
**13**	Soft dough	2×	1×	0
**17**	Dent	1×	0	0
**Total irrigation applied (mm)**		64.5	43.68	0.0

* Number of irrigation events. Note: Total rainfall for (2022: 453.14 mm) and (2023: 568.96 mm) May to September.

## Data Availability

Data are contained within the article.

## Abbreviations

ANOVA: analysis of variance; DAP: days after planting; HI: high number of irrigation events; LO: low number of irrigation events; LWP: leaf water potential; MREC: Milan Research and Education Center; RF: rainfed; RWC: relative water content; VPD: vapor pressure deficit; VRI: variable rate irrigation; WAS: weeks after sowing.
